# Hyaluronic Acid and Calcium Hydroxyapatite in the Context of Hypertrophic Photoaging. Evaluation by 2D, 3D Photographs and Reflectance Confocal Microscopy (RCM)

**DOI:** 10.1111/jocd.16605

**Published:** 2024-09-27

**Authors:** Ilaria Proietti, Federica Trovato, Francesca Paola Sasso, Emanuele Amore, Concetta Potenza, Stefania Guida, Giovanni Pellacani

**Affiliations:** ^1^ Dermatology Unit Daniele Innocenzi A. Fiorini Hospital Terracina Italy; ^2^ Dermatology Unit, Department of Clinical Internal, Anesthesiological and Cardiovascular Science University of La Sapienza Rome Italy; ^3^ Dermatology Department IDI–Istituto Dermopatico dell'Immacolata IRCCS Roma Italy; ^4^ Dermatology Clinic IRCCS San Raffaele Hospital Milan Italy; ^5^ Faculty of Medicine Vita‐Salute San Raffaele University Milan Italy

**Keywords:** calcium hydroxyapatite, collagen, hyaluronic acid, hybrid filler, hypertrophic skin photoaging

To the Editor,

Our understanding of injectables has changed a lot from the 1990s to today. In the past we were looking for the near‐ideal filler and discovering the advantages of temporary fillers. Nowadays we are looking for injectables that emulate the physiology of regeneration and we are discovering the effects of injectables on skin regeneration [1]. Biomaterial‐based injectables for anti‐aging and rejuvenation purposes have long been used in regenerative and aesthetic medicine. The injection induces a phlogistic response that causes a series of processes, ranging from tissue regeneration to fibrosis [2, 3]. These are aesthetic regenerative scaffolds: injected biomaterials that can predetermine the inflammatory response, inhibiting chronic inflammatory response, reverting fibrosis, and enhancing physiological tissue regeneration. They include calcium hydroxyapatite (CaHA), hyaluronic acid (HA), and poly‐L‐lactic acid (PLLA) dermal fillers [4].

We present the case of a 56‐year‐old woman with Fitzpatrick skin phototype III, who complained of prominent wrinkles, dull facial appearance, and uneven skin texture. She denied smoking habit, reported spending a lot of time outdoors for work purposes and never applying sunscreen. On clinical observation, the patient displayed all the features of hypertrophic photo‐aging (HP) (Figure 1A). HP is characterized by deep wrinkles and a leathery appearance of the skin, primarily affecting skin phototypes III–IV. HP presents clinically with responses such as permanent tan, deep wrinkles, coarseness, leathery skin, that correspond to histological feature as reduced epidermal thickness, reduced CD44 expression, elastosis, reduced amount of elastic fibers, loss of fibrillin‐rich microfibrils (FRMs) at the dermo‐epidermal junction (DEJ), aberrant arrangement of collagen in dermis, reduced intensity of collagen VII [5, 6].

**FIGURE 1:**
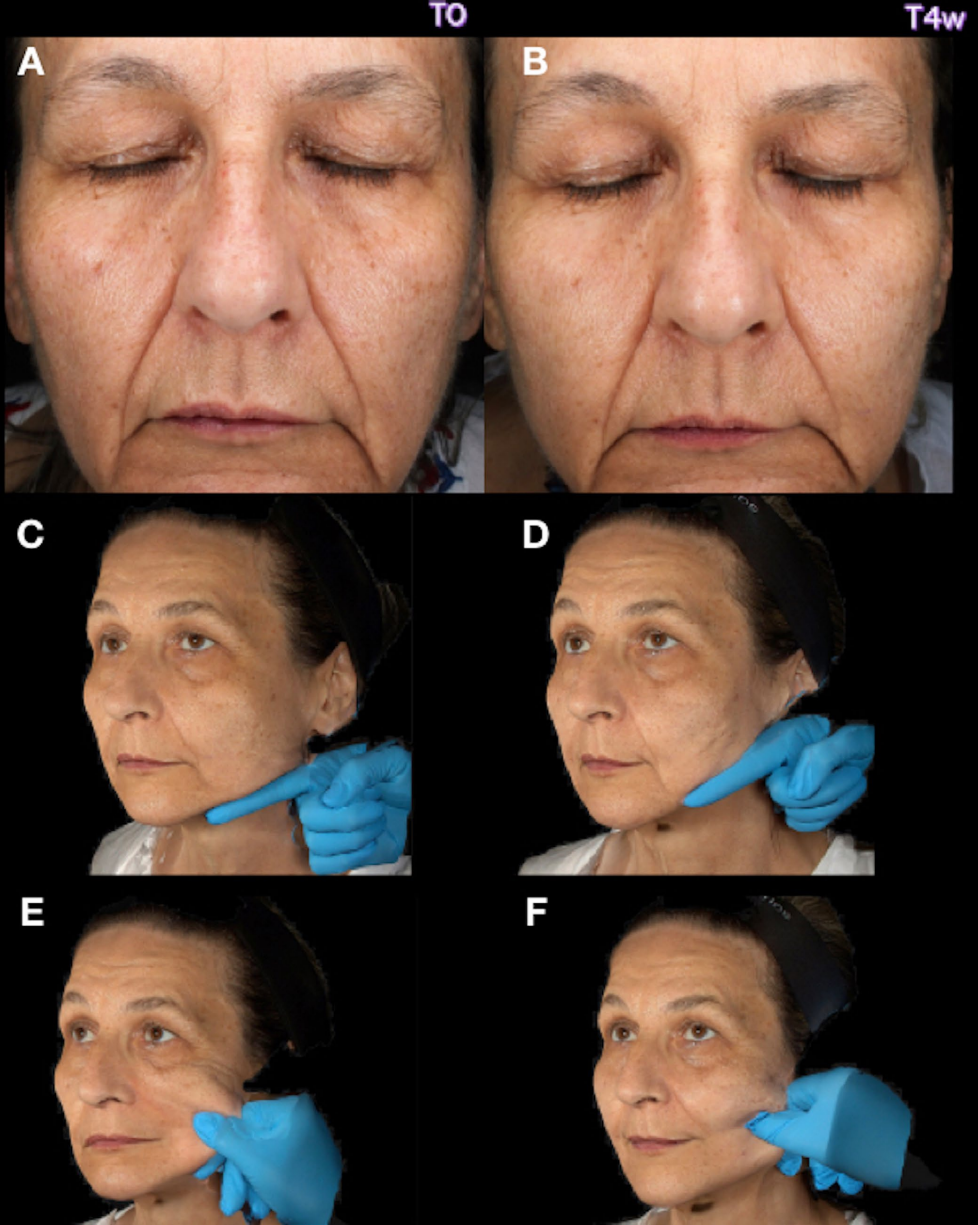
Two‐dimension pictures (A) at T0 and (B) T4w. Corresponding three‐dimension pictures comparisons: (C) finger test at T0 versus (D) at T4w and (E) pinch test at T0 versus (F) at T4w.

We decided to use a hybrid injectable, combining HA and CaHA, in a 1.25 mL prefilled syringe with hydrochloride lidocaine (3 mg/mL). The patient was injected with cannula 22G 70 mm, 1 syringe per each side, at T0, and a follow‐up visit was scheduled after 4 weeks (T4w). At T0 and T4w we obtained two‐dimensional (2D) and three‐dimensional (3D) photos, and reflectance confocal microscopy (RCM) Figures 1 and 2.

**FIGURE 2:**
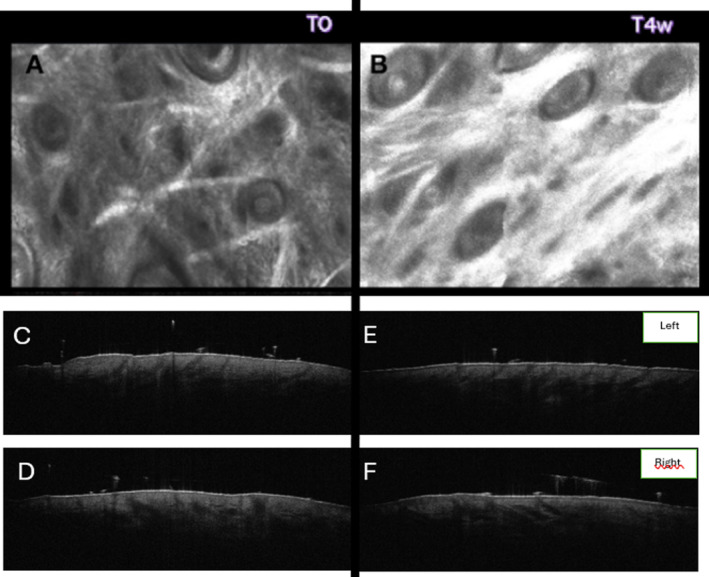
Reflectance confocal microscopy images (A) at T0 showing fragmented hyporefractive collagen fibers at dermo‐epidermal junction and upper dermis (B) and at T4w highlighting hyperrefractive collagen fibers organized in a net. OCT images with increased dermal density and collagen deposition from baseline (T0) (C and D) to Week 4 (T1) (E and F). Collagen fibers are more compact with a uniform and sustained structure.

The comparison of 2D and 3D pictures at T0 and T4w highlighted a bright appearance of the skin, reduced wrinkles depth, improved texture, and increased tissue support after treatment Figures 1 and 2. Specifically, 3D assessment provided an even better evaluation of the restored facial structure with improved skin firmness and elasticity, as confirmed by finger and pinch tests (Figure 1C–E).

Additionally, RCM at the level of DEJ/superficial dermis revealed a hyporefractive and fragmented coarse network of collagen (Figure 2A) at T0 while hyperrefractive collagen fibers organized in linear network were observed at T4w (Figure 2B). The optical coherence tomography (OCT) analysis conducted on the left and tight cheeks at T0 and T1 revealed an improvement in collagen disposition. Collagen density increased after 4 weeks (Table 1; Figure 2C–F). This experience aims to share our decision‐making paths in the field of injectables. The choice of injectables in the field of facial rejuvenation has to be based on the needs of the patient's skin. In this case, our patient needed the rearrangement of fibrillar collagen fibers of papillary dermis in linear and ordered pattern, the reconstitution of the structure of the skin, and the regeneration of the DEJ. We chose a HA + CaHA‐based injectable to induce fibroblast differentiation, stimulate neocollagenogenesis, and restore plumpness, as supported by clinical results and non‐invasive skin imaging. We firmly believe that within the plethora of fillers available, choosing the right product for the right patient is critically important for a successful treatment.

**TABLE 1:** ROI1 statistics.

	Left cheek	Right cheek
Density (T0)	3 642 852	38,041395
Density (T1)	4 817 985	49,297926
Attenuation (T0)	0,002323	0,002596
Attenuation (T1)	0,003104	0,002971

## Ethics Statement

The study was conducted in accordance with the Declaration of Helsinki.

## Consent

Informed consent was obtained from subjects involved in the study.

## Conflicts of Interest

The authors declare no conflicts of interest.

## Data Availability

The data that support the findings of this study are available from the corresponding author upon reasonable request.
